# Hypertension Prevalence Rates Among Urban and Rural Older Adults of China, 1991–2015: A Standardization and Decomposition Analysis

**DOI:** 10.3389/fpubh.2021.713730

**Published:** 2021-09-17

**Authors:** Qi Yu, Shiqi Lin, Jilei Wu

**Affiliations:** Institute of Population Research, Peking University, Beijing, China

**Keywords:** the elderly, urban and rural, regional difference, standardization and decomposition analysis, trend

## Abstract

**Objectives:** The prevalence of hypertension (HTN) among older adults is becoming an important issue in public health in China as it is now stepping into the super-aged society with high pressure of a chronic disease burden. With urban–rural differences in population composition and health facilities, this study aimed to assess the gaps in the prevalence trends of HTN among older adults by considering demographic factors such as age, gender, education level, and regional differences during 1991–2015 in China.

**Methods:** We adopted the consistent sampling design and measure of HTN of the cross-longitudinal surveys of the China Health and Nutrition survey, and we compared the HTN prevalence rates between urban and rural older adults by taking each wave of the survey as a cross-sectional sample of the Chinese population by the following and supplementary samples. The classic standardization and decomposition analysis method was utilized with four factor-specific rates, and contributions were calculated, i.e., age, gender, education, and region, which reflects the aspect of demographic and social development differences between urban and rural areas of China.

**Results:** The prevalence rates of HTN of the whole of older adults were increasing in 1991–2015. However, the gaps of prevalence rates of HTN between urban and rural areas show different trends accompanied by the health policies launched by the government. Namely, the gap was narrowed during 1993–1997 and then enlarged during 1997–2011 and narrowing again. Those trends reflect the policy effects with the health resource allocation and utilization of health services for urban and rural older adults.

**Conclusions:** With the four factors of decomposition analysis, the differences reflect the results of health policy effects, considering the urban–rural discrepancy on older adults with different demographic characteristics. Hence, the differentiated policies should be considered with the urban–rural population, such as HTN prevention and the population health promotion.

## Introduction

Hypertension (HTN), as a chronic medical condition in which the blood pressure in the arteries is elevated, is becoming a major threat to population health, especially in older people accompanied with other chronic diseases. The economic burden of disease caused by HTN accounted for 7.0% of the global disability-adjusted life years (DALYs) in 2010 (1). With the acceleration of the aging process, China is facing a very serious situation of HTN. Since the beginning of this century, more and more investigations on HTN have been conducted nationwide. From 1991 to 2011, the standardized prevalence of HTN in adults aged 18 years and above increased from 23.9 to 33.6% (2). The prevalence of HTN of older adults (aged 60 years old and over) was 54.6% (3), which was significantly higher than that in the young population (2).

In developed countries, 60% of individuals after age 65 suffer from HTN (4). As one of the largest developing countries, China is challenged even more by HTN as a growing urgency in public health. Due to its long-term urban–rural dual social structure, the prevalence of HTN in urban and rural areas have been different for a long time. Some studies suggest that the prevalence of HTN in rural adults is higher than in urban areas (5). Yet other studies have found different results. There is some evidence that HTN is very common among urban Chinese (6). Between 1993 and 2011, the HTN rate in urban areas was higher than in rural areas, and the rural–urban gaps were narrowed from 7.75 to 1.12% for adults (5). The prevalence of HTN in rural areas was 34.3 and 63.9% in urban areas for the aged in 2013 (7). These inconsistent results mean further investigation is needed, especially among the older population.

However, when comparing HTN rates among older people or exploring temporal trends within a period, the results are usually influenced by the composition of the target population, especially age structure, gender proportion, education levels, and other demographic-social factors, which reveal different exposure factors for HTN. Thus, factor standardization on the rates is necessary. Taking age as an example, there is a significant difference in whether the prevalence of HTN was age-standardized or not. With age-standardization, the prevalence of HTN was 14.0 and 15.3% for crude and age standardization in 1991 although the crude and age standardization rates were 34.1 and 25.6%, respectively, in 2015 (8). Without the age standardization, the crude prevalence rates of HTN are overestimated or underestimated. Standardization by demographic and sociological factors is essential when estimating the prevalence of HTN and policy making on prevention.

As crude rates cannot be directly compared in two or more populations without standardization, there are two ways of obtaining the “true rate,” i.e., the standardization rate, which excludes the effects of population structures. One is to perform decomposition on cross-classified data, which involves one or more factors, and the other can be expressed as a function of two or more factors; decomposes the rates into composition and the composition-specific rate (9–11). The HTN rates of older adults in urban and rural areas can be decomposed and standardized by considering the different structures of two populations. Then, we can get the “true” HTN rates to compare and evaluate each factor's contribution, and then find out the targeting policy on prevention of HTN among older adults.

The prevalence of HTN is affected by social-economic situation and individual behavior factors. The former usually relates to social development and health resources allocation and utilization among different areas of people, and the latter mainly includes personal demographic characteristics and behavior variables, such as income situation, smoking, alcohol consumption, and others. We selected demographic and social development factors, including age, gender, and urban/rural as well as education level as the composition factors of the population and measure their effects by decomposition.

Facing the social-economic development gaps between urban and rural areas in China, the changes in age distribution may be more pronounced for older populations in urban areas. Besides this, age is the basic character of the population that influences all the other factors we analyze—gender, education, and region—all the other factors are disaggregated by age. There are significant gender differences in the prevalence of HTN. The prevalence of HTN in males is significantly higher than in females due to factors such as genes, hormones, lifestyle, and psycho-social factors with age standardization (8). However, people in urban areas tend to face more work pressure and eat more fatty food in their daily life than the people in rural areas, especially with fast urbanizing in China. As a result, the accumulation of risk factors can lead to the higher prevalence of HTN among urban older adults than rural. For educational attainment, different educational levels are usually related to knowledge of HTN and then the prevention and control of it. With higher education levels, people are well-educated and increase their knowledge of health behaviors, and HTN patients among older adults are expected to decline, especially for rural areas. The region represents the different living habits and economic development, which are related to health resources allocation and utility of health services. For example, people in the central region have a relatively high intake of oil and salt: Shaanxi was 17.9 g and Guangxi was 7.6 g daily salt intake (12). Great changes have taken place with economic development in China, and to some extent, it reflects the process of urbanization and medical and health care. However, there are still obvious regional differences in China's economic development, notably the rapid development in the east and the relatively backward development in the west. There are also obvious regional differences between urban and rural areas.

To sum up, when studying the urban–rural gaps in the prevalence of HTN and temporal trends, most previous studies focus on all Chinese adults with less specific focus on older adults in an aging society. It is not clear to what extent the rural–urban gap exists in the prevalence of HTN among Chinese older adults, especially in terms of crude prevalence. This may confuse the prevalence of HTN among older adults and is not conducive to identifying the influence of different demographic factors on HTN. Because China has social differences between urban and rural and social and economic development, the HTN rates among older adults in those areas may have different characteristics. Most studies directly compare crude rates of HTN, which is a major drawback. We focus on the population factors—age, gender, education, and region—aiming to identify the influencing factors on the prevalence of HTN, utilizing 1991–2015 China Health and Nutrition Survey data. We compare the HTN rates between urban and rural older adults over time, and further discuss the main factors influencing the urban–rural gaps to help policymakers develop more targeted public health policies to address the health differences between urban and rural older adults.

## Methods

### Data

This study used data from the China Health and Nutrition Survey (CHNS), which is a national survey on nutritional status, health risk factors, and health status among people aged over 2 years in China. The CHNS has conducted 10 waves (1989, 1991, 1993, 1997, 2000, 2004, 2006, 2009, 2011, and 2015) and is designed to provide representation of urban, suburban, and rural areas. It employs a multistage, random-cluster process to ensure good representation of the general Chinese population (13). It selects samples by province that vary in socioeconomic development and geography in each wave (14) and focuses on health during urbanization and economic development (15).

Although the CHNS was a cross-longitudinal survey, this study took each wave as a cross-sectional survey. There are nine waves of sampled data (1991, 1993, 1997, 2000, 2004, 2006, 2009, 2011, and 2015) analyzed, taking advantage of the fact that CHNS surveys employ a unified sampling and HTN measurement criteria, and new samples in each wave to ensure representative coverage for the Chinese. We calculated the prevalence and time trend of HTN for older adults (age 60 and above) after excluding missing data.

### Variables

The HTN rates were calculated with the numerator as the number of respondents diagnosed with HTN by doctors and the denominator as the total number of respondents in a survey year. Participants in the survey were also measured three times at 1- to 2-min intervals to get systolic (SBP) and diastolic blood pressure (DBP) with 5 min rest. Thus, in cases of missing diagnosis information, the mean values of SBP and DBP were obtained from three measurements. An individual is considered hypertensive if the person's SBP ≥ 90 or DBP ≥140.

The responses were divided into three groups by age as 60–69, 70–79, and 80+ years old. Education level is divided into uneducated and educated according to whether or not they went to school. We grouped 15 provinces into three regions: east (Beijing, Liaoning, Jiangsu, Shandong, Zhejiang, and Shanghai), central (Heilongjiang, Henan, Hubei, and Hunan), and west (Yunnan, Chongqing, Guangxi, Guizhou, and Shaanxi).

### Standardization and Decomposition Method

The decomposition method (10, 11, 16) was used to identify the changes of HTN rates and the attributes of demographic and social development compositions, including age, gender, education, and region. Both the standardization and decomposition analysis were calculated on rural and urban older adults separately. The algebraic formula for two-population standardization and decomposition analysis with four factors can be expressed as the following steps.

First, the crude HTN rate for rural and urban older adults is equal to the total number of people who have HTN divided by the total number of older adults in that population. The crude rates can be expressed as


(1) $$\begin{array}{r} T_{....}=\underset{ijkl}{\sum }\frac{T_{ijkl}N_{ijkl}}{N_{....}}, \end{array}$$

(2) $$\begin{array}{r} t_{....}=\underset{ijkl}{\sum }\frac{t_{ijkl}n_{ijkl}}{n_{....}}. \end{array}$$

There are four factors: *i*, *j*, *k*, and *l*, which are age, gender, region, and education; *N*_...._ and *T*_...._ are the number of older adults and the crude HTN rate in urban areas; *N*_*ijkl*_ and *N*_*ijkl*_ denote the number and HTN rate of older adults for the (*i*,*j*,*k*,*l*) categories in urban areas in formula (1). Similarly, the crude HTN rate in rural areas can be seen in formula (2).

In formulas (1) and (2), *N*_*ijkl*_/*N*_...._ and *n*_*ijkl*_/*n*_...._ can be written as


$$\begin{array}{l} \frac{N_{ijkl}}{N_{....}}=A_{ijkl}B_{ijkl}C_{ijkl}D_{ijkl}, \end{array}$$

where


(3) $$\begin{array}{r} \begin{array}{c} A_{ijkl}={\left(\frac{N_{ijkl}}{N_{.jkl}}\right)}^{\frac{1}{4}}{\left(\frac{N_{ijk.}}{N_{.jk.}}\;\cdot \;\frac{N_{ij.l}}{N_{.j.l}}\;\cdot \;\frac{N_{i.kl}}{N_{..kl}}\right)}^{\frac{1}{12}}{\left(\frac{N_{i..l}}{N_{...l}}\;\cdot \;\frac{N_{i.k.}}{N_{..k.}}\;\cdot \;\frac{N_{ij..}}{N_{.j..}}\right)}^{\frac{1}{12}}{\left(\frac{N_{i...}}{N_{....}}\right)}^{\frac{1}{4}}\\ B_{ijkl}={\left(\frac{N_{ijkl}}{N_{i.kl}}\right)}^{\frac{1}{4}}{\left(\frac{N_{ijk.}}{N_{i.k.}}\;\cdot \;\frac{N_{ij.l}}{N_{i..l}}\;\cdot \;\frac{N_{.jkl}}{N_{..kl}}\right)}^{\frac{1}{12}}{\left(\frac{N_{.j.l}}{N_{...l}}\;\cdot \;\frac{N_{.jk.}}{N_{..k.}}\;\cdot \;\frac{N_{ij..}}{N_{.i..}}\right)}^{\frac{1}{12}}{\left(\frac{N_{.j..}}{N_{....}}\right)}^{\frac{1}{4}},\\ C_{ijkl}={\left(\frac{N_{ijkl}}{N_{ij.l}}\right)}^{\frac{1}{4}}{\left(\frac{N_{ijk.}}{N_{ij..}}\;\cdot \;\frac{N_{i.kl}}{N_{i..l}}\;\cdot \;\frac{N_{.jkl}}{N_{.j.l}}\right)}^{\frac{1}{12}}{\left(\frac{N_{..kl}}{N_{...l}}\;\cdot \;\frac{N_{.jk.}}{N_{..j.}}\;\cdot \;\frac{N_{i.k.}}{N_{.i..}}\right)}^{\frac{1}{12}}{\left(\frac{N_{..k.}}{N_{....}}\right)}^{\frac{1}{4}}\\ D_{ijkl}={\left(\frac{N_{ijkl}}{N_{ijk.}}\right)}^{\frac{1}{4}}{\left(\frac{N_{ij.l}}{N_{ij..}}\;\cdot \;\frac{N_{i.kl}}{N_{i..k}}\;\cdot \;\frac{N_{.jkl}}{N_{.j.k}}\right)}^{\frac{1}{12}}{\left(\frac{N_{..kl}}{N_{...k}}\;\cdot \;\frac{N_{.j.l}}{N_{..j.}}\;\cdot \;\frac{N_{i..l}}{N_{.i..}}\right)}^{\frac{1}{12}}{\left(\frac{N_{...l}}{N_{....}}\right)}^{\frac{1}{4}} \end{array} \end{array}$$

where *A*_*ijkl*_, *B*_*ijkl*_, *C*_*ijkl*_, and *D*_*ijkl*_ denote the (age, gender, region, and education) category distribution for urban older adults. The ratio *n*_*ijkl*_/*n*_...._ can be calculated in a similar way by using lowercase a, b, c, d, and n. Then, the differences of older adults' HTN rates between the rural and urban population are as follows:


(4) $$\begin{array}{l} T_{2..}-T_{1..}=I-effect+J-effect+K-effect+L-effect\\ \;+Rate\;effect\\ \;=\left[R\left(\bar{T}\right)-R\left(\bar{t}\right)\right]+\left[I\left(\bar{A}\right)-I\left(\bar{a}\right)\right]+\left[J\left(\bar{B}\right)-J\left(\bar{b}\right)\right]\\ \;+\left[K\left(\bar{C}\right)-K\left(\bar{c}\right)\right]+\left[L\left(\bar{D}\right)-L\left(\bar{d}\right)\right]. \end{array}$$

Standardization rates for older adults in urban areas can be calculated as follows:


(5) $$\begin{array}{l} R\left(\bar{T}\right)=\left(I,J,K,L\right)-\text{standardized rate}=\underset{ijkl}{\sum }\frac{\frac{N_{ijkl}}{N_{....}}+\frac{n_{ijkl}}{n_{....}}}{2}T_{ijkl}\\ \begin{array}{c} I\left(\bar{A}\right)=\left(J,K,L,R\right)-\text{standardized rate}=\underset{ijkl}{\sum }\frac{T_{ijkl}+t_{ijkl}}{2}\\ J\left(\bar{B}\right)=\left(I,K,L,R\right)-\text{standardized rate}=\underset{ijkl}{\sum }\frac{T_{ijkl}+t_{ijkl}}{2}\\ K\left(\bar{C}\right)=\left(I,J,L,R\right)-\text{standardized rate}=\underset{ijkl}{\sum }\frac{T_{ijkl}+t_{ijkl}}{2}\\ L\left(\bar{D}\right)=\left(I,J,K,R\right)-\text{standardized rate}=\underset{ijkl}{\sum }\frac{T_{ijkl}+t_{ijkl}}{2} \end{array}. \end{array}$$

The standardized rates $R\left(\bar{T}\right)$, $I\left(\bar{A}\right)$, $J\left(\bar{B}\right)$, $K\left(\bar{C}\right)$, and $L\left(\bar{D}\right)$ are the rate effect and (age, gender, region, and education) effect for urban older adults, respectively. We can also obtain $R\left(\bar{t}\right)$, $I\left(\bar{a}\right)$, $J\left(\bar{b}\right)$, $K\left(\bar{c}\right)$, and $L\left(\bar{d}\right)$ by replacing the letters in (5) for the rural population.

Besides this, the bootstrap resample was used to estimate the standard errors and confidence intervals for the component effects, which were based on 100 bootstrap resamples (16). Taking the ${\hat{\sigma }}_{\hat{\theta }}$ as the component effect estimated by bootstrapping resamples, the distribution of ${\hat{\theta }}_{b}$ represents the sample distribution of component effects, and ${\hat{\theta }}_{\left(.\right)}$ denotes the mean value of ${\hat{\theta }}_{b}$. Then, the standard deviation of ${\hat{\theta }}_{b}$ value is


(6) $$\begin{array}{l} {\hat{\sigma }}_{\hat{\theta }}={\left\{\left[\sum {\left({\hat{\theta }}_{b}-{\hat{\theta }}_{\left(.\right)}\right)}^{2}\right]/\left(B-1\right)\right\}}^{1/2}.\\ {\hat{\theta }}_{\left(.\right)}=\sum {\hat{\theta }}_{b}/B \end{array}$$

In this way, we can get the standardized HTN rates and the factor effect to compare the “true rate” between the two populations and identify the most important influencing factor for each year.

## Results

**Figure 1:**
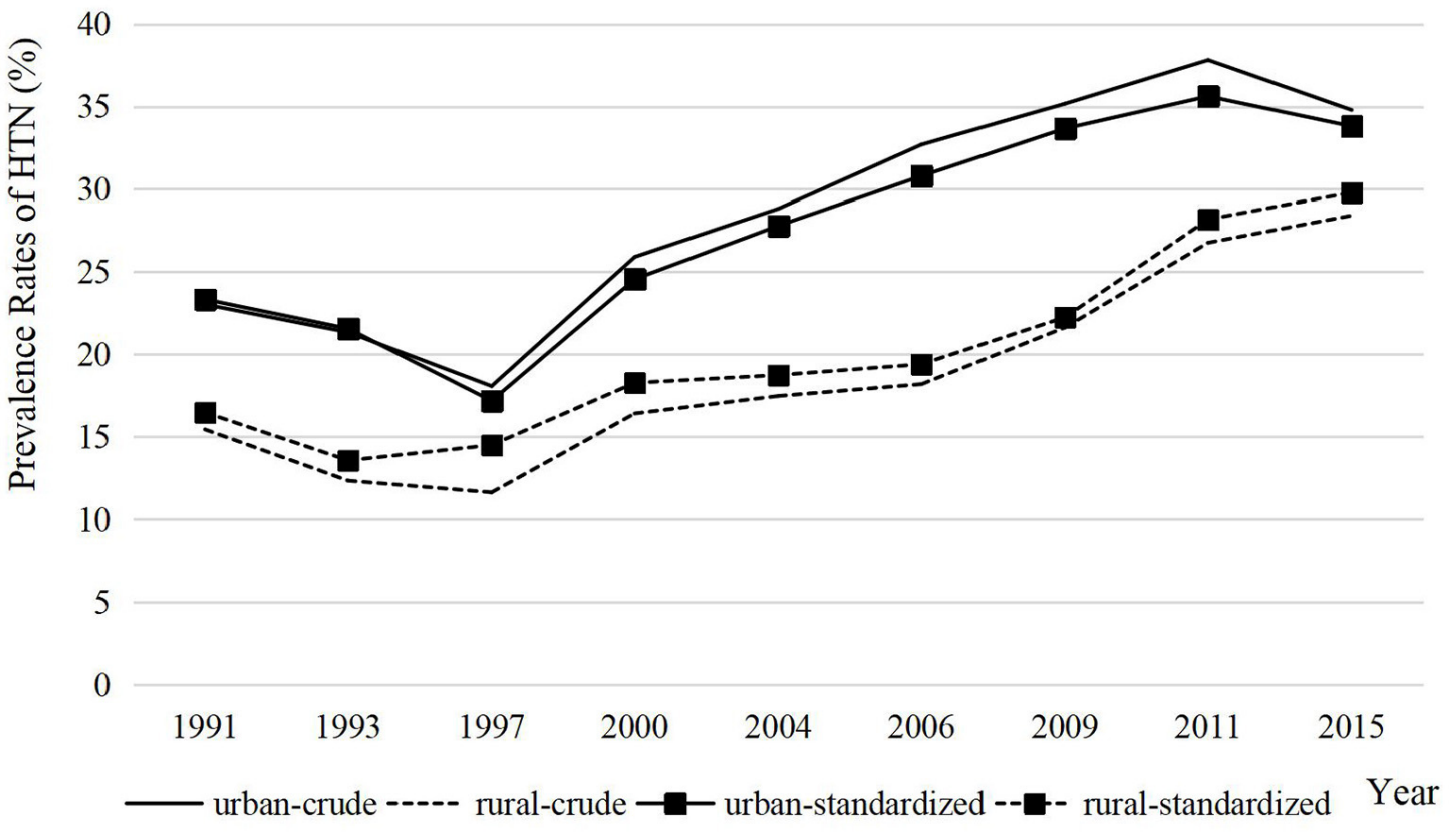
Crude and standardized prevalence rates of HTN in older adults in urban and rural areas, 1991–2015.

The crude and standardized HTN rates of urban and rural older adults during 1991–2015 are plotted as Figure 1. There are three characteristics during this period. First, the HTN rates of Chinese older adults increased in 1991–2015 with the HTN rates in urban areas being higher than in rural areas throughout the period. Second, the gaps between urban and rural areas showed a dynamic change characteristic with a decreasing trend during 1993–1997, an expanding trend during 1997–2011, and a decreasing trend during 2011–2015 again. Third, the gaps for HTN area rates were narrower with standardization than without over the entire period, especially in 1997 when the gap was only about 2% with standardization. It illustrates that direct calculation of crude rates would exaggerate the urban–rural gaps.

The decomposition results of demographic and social development factors (age, gender, education, and region) are shown in Table 1, and it reveals two facts of prevalence of HTN. First, all the factors contributed to the trend among older adult HTN rates over 1991–2015, and almost all factor-specific differences were statistically significant. In particular, region had the biggest impact on HTN rates of older adults, and the influence on the urban population was significantly greater than rural. This fact indicates that the regional difference was the main factor driving the trend change of HTN rates of older adults in urban and rural areas during the period. For example, the contribution of region to older adult HTN rates in urban areas was 32.38 and 30.46% in rural areas in 2015 with a statistical significance difference (1.92%). Second, the standardization rates of each factor showed two stages for both areas during this period. Namely, the factor-specific standardization rates showed a downward trend during 1991–1997 and an upward trend in 1997–2015.

**Table 1:** Results of standardization HTN rates (%) and gaps between urban and rural areas of the older adults by demographic and social development factors, 1991–2015.

		***N***	**Age**	**Gender**	**Education**	**Region**	**Standardized rates**	**Crude rates**
1991	Urban	*n* = 343	19.26	19.14	19.31	19.26	23.34	23.03
	Rural	*n* = 349	18.97	19.21	19.03	19.04	16.47	15.44
	Gap		0.29^*^	−0.07	0.28^*^	0.22^*^	6.87^*^	7.59^*^
1993	Urban	*n* = 321	16.82	16.58	17.13	16.42	21.56	21.34
	Rural	*n* = 403	16.36	16.65	16.13	16.79	13.56	12.33
	Gap		0.46^*^	−0.07	1^*^	−0.37^*^	8^*^	9.01^*^
1997	Urban	*n* = 461	15.36	15.17	15.92	15.51	17.18	18.07
	Rural	*n* = 575	14.62	14.99	14.1	14.49	14.49	11.62
	Gap		0.74^*^	0.18^*^	1.82^*^	1.02^*^	2.69^*^	6.44^*^
2000	Urban	*n* = 525	21.02	20.85	21.37	21.59	24.56	25.91
	Rural	*n* = 683	20.63	20.8	20.2	19.99	18.27	16.41
	Gap		0.39^*^	0.05	1.17^*^	1.6^*^	6.29^*^	9.5^*^
2004	Urban	*n* = 729	23.13	23.07	23.03	23.89	27.79	28.82
	RURAL	*n* = 1,052	22.88	22.91	22.93	22.09	18.74	17.47
	Gap		0.25^*^	0.16^*^	0.1	1.8^*^	9.05^*^	11.35^*^
2006	Urban	*n* = 803	25.16	24.86	25.09	25.49	30.8	32.7
	Rural	*n* = 1,242	24.36	24.68	24.43	24.02	19.4	18.19
	Gap		0.8^*^	0.18^*^	0.66^*^	1.47^*^	11.4^*^	14.51^*^
2009	Urban	*n* = 937	27.9	27.77	27.64	28.58	33.67	35.17
	Rural	*n* = 1,584	27.51	27.67	27.78	26.82	22.25	21.63
	Gap		0.39^*^	0.1	−0.14^*^	1.76^*^	11.42^*^	13.54^*^
2011	Urban	*n* = 1,563	31.73	31.67	31.87	32.78	35.62	37.82
	Rural	*n* = 2,072	31.39	31.49	31.24	30.32	28.18	26.77
	Gap		0.34^*^	0.18^*^	0.63^*^	2.46^*^	7.44^*^	11.04^*^
2015	Urban	*n* = 1,981	31.54	31.45	31.61	32.38	33.81	34.79
	Rural	*n* = 2,752	31.38	31.51	31.25	30.46	29.8	28.41
	Gap		0.16^*^	−0.06	0.36^*^	1.92^*^	4.01^*^	6.38^*^

* *P < 0.05*.

**Figure 2:**
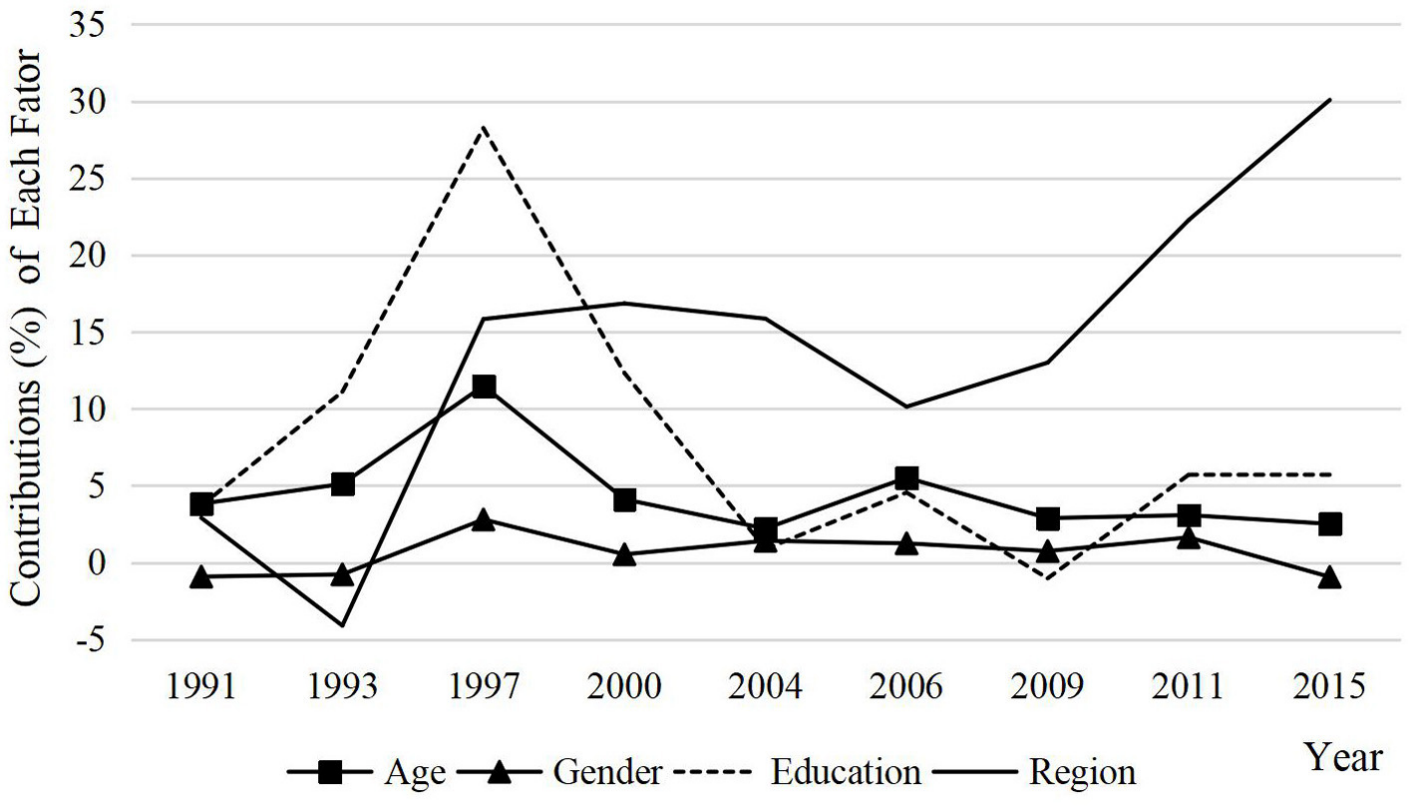
Contributions (%) of each factor in HTN rate of older adults by decomposition, 1991–2015.

Table 2 presents each factor's varying contributions to older adult HTN rates during 1991–2015 in both urban and rural areas. To facilitate understanding, we used a plot to exhibit the contribution differences among each factor (Figure 2). The plus and minus signs represent directions of factors. For example, the contribution of gender effect in 1991 was −0.9222, meaning the gender effect tended to narrow the urban–rural gaps of older adults' HTN rates. Similarly, the contribution of age effect in 1991 was 3.8205, representing that age increased the urban–rural gap.

**Table 2:** Contributions (%) of each factor in HTN rates of older adults by decomposition, 1991–2015.

	**Age**	**Gender**	**Education**	**Region**	**Standardized rates**	**Crude rates**
1991	3.8205	−0.9222	3.6887	2.8983	90.5054	99.9907
1993	5.1071	−0.7772	11.1024	−4.1079	88.8192	100.0327
1997	11.4855	2.7938	28.2482	15.8314	41.7514	99.955
2000	4.1069	0.5265	12.3208	16.849	66.2375	100.0408
2004	2.2024	1.4095	0.881	15.8574	79.7277	99.9899
2006	5.5137	1.2406	4.5488	10.1313	78.5697	100.004
2009	2.8811	0.7387	−1.0342	13.0017	84.3634	100.0245
2011	3.0785	1.6298	5.7044	22.2742	67.3658	99.9622
2015	2.5072	−0.9402	5.6412	30.0863	62.8366	99.9744

Two main discoveries could be found through Table 2 and Figure 2. First, all the factors have contributions to older adults HTN rates, and almost factors had a positive effect on HTN rates and tended to enlarge the urban–rural gap. Second, by comparing the contributions of factors in different years, on the whole, region contributed the most, age and education were also relatively important, and gender was the least. In addition, as time went by, the region effect contributed more and more, especially in 2009 and beyond, and the contributions of the other three factors showed a steady trend. This means that the influence of regional differences on HTN rates is becoming more and more obvious by comparison with other factors, which is also the focus of regional prevention and control of HTN for older adults in the future.

## Discussion

With the acceleration of the aging process, the prevalence of HTN and the economic burden of the disease caused by HTN among older adults are increasing. Due to the influence of confounding factors of social and demographic components, the traditional crude rates cannot truly reflect the distribution characteristics of HTN. In this case, it is necessary to standardize and decompose the HTN rates of older adults to identify the prevalence trend and distribution characteristics of HTN and to clarify the most critical affecting factors. These will help policymakers to formulate more targeted and accurate policies for the prevention and control of HTN.

The study focused on how social and demographic factors affected the trend of HTN rates among urban and rural older adults by a standardizing and decomposing method. First, our results reveal an increasing trend of HTN rates for Chinese older adults in 1991–2015, and the HTN rates in urban areas were higher than the rural. In many developing countries, the prevalence of HTN is going upward, including China, especially with the acceleration of the aging society (17). National-level research found that nearly half of 35- to 75-year-old adults in China had HTN (18), and the rates are still increasing rapidly in recent years (19). With age- and sex-standardization, the HTN rates have increased from 60.1% to 65.2% from 2001 to 2010 in urban areas (19). However, the rates were consistently found to be higher in urban areas than rural areas throughout 1991–2015 as some previous studies in the 1990s and 2010 prove (20–22). Two reasons might help explain those findings. On one hand, compared with rural areas, the occupational pressure and interpersonal relationships in urban areas are more intense, so are unhealthy lifestyles, which are all important risk factors of HTN. On the other hand, as confirmed in some studies (5), the accessibility and utilization of health services in cities are much higher, and the awareness of health care is stronger. Therefore, the detection rates of HTN are higher. This also suggests that the relatively lower HTN rates in rural areas may also be caused by insufficient medical and health resources, which still needs further verification.

Second, we found that HTN rates of urban–rural gaps for older adults showed a dynamic change trend, and this helps us to identify temporal factors that influence the prevalence of HTN by standardization. We found that the urban–rural gaps experienced a decreasing trend during 1993–1997, then increased in 1997–2011, and decreased in 2011–2015 again. The dynamic change trend of the urban–rural gaps in 1997–2015 exactly show that the Chinese government has been issuing relevant policies to promote urban–rural equity. We conclude that this trend was mainly realized by adjusting medical insurance policies. Basic Medical Insurance for Urban Employees has been carried out in some enterprises since 1988 and was officially launched nationwide in 1998. As compulsory insurance, it covered all employees in urban areas. Before 1998, the urban–rural gap in HTN was relatively narrow. Taking 1997 as the inflection point, older adults living in urban areas may enjoy preferential policies to improve the utilization of medical and health services, which promotes HTN prevention to a certain extent. As a result, after the implementation of the medical insurance policy in cities, the urban–rural gaps show a significant trend of expansion. However, since the New Rural Cooperative Medical scheme was adjusted around 2011, the gaps between urban and rural areas of HTN changed accordingly. The New Rural Cooperative Medical scheme was implemented for rural residents in 2003 and had basically covered all rural residents by 2010. This study concludes that the coverage of medical insurance policies for the rural population has greatly improved the economic security of medical care for them. Therefore, the urban–rural gaps of HTN took 2011 as the turning point, showed a large trend before 2011, decreasing after that. This dynamic change trend coincides with the change of the medical insurance policy in China, which reflects the important role of medical insurance policies in the prevention of HTN. However, other studies also using China Health and Nutrition Survey data have found that the urban–rural gaps of HTN prevalence were gradually narrowed during 1993–2011 (5). We consider that the difference between the two results is most likely due to whether the HTN rates were standardized. Because the composition of populations may be different, it is necessary to standardize rates when we compare two or more populations. Standardized rates give us an opportunity to understand the true rates of HTN and identify the period factors of urban–rural gaps, namely, medical insurance policies. This also shows that a medical insurance policy is an important measure to promote health equity for older adults in both urban and rural areas. It can provide economic security, improve the utilization of medical and health services, and carry out early prevention and intervention of diseases. Therefore, an important way to prevent and treat HTN is to provide strong economic support for medical treatment, especially for the rural areas. For example, the reimbursement ratio should be increased, and more HTN prevention and treatment drugs should be included in the reimbursement scope to promote the health equity of the older adults in urban and rural areas.

Third, comparing different compositions, region was the most crucial driving force in the trends of older adults' HTN rates with an ever-rising impact over time. Although age, gender, and education level were all influencing factors for HTN, their effects were relatively limited and stable in recent years. This result is different from other studies, which have found that there are significant statistical differences in the effect of demographic compositions, such as age, gender, and education, on HTN rates (23). However, this is not a contradiction. We do not deny the differential influence of demographic factors on the prevalence of HTN. Whereas, with the gradual adaptation of society to aging and urbanization as well as the improvement of education levels, the influence of demographic factors, such as age and education, on HTN tends to be stable. Instead, social development factors might be making more important contributions. Due to the dualistic social structure that has existed for a long time, urban–rural gaps are typical representatives of socioeconomic differences in China, which further lead to other differences, such as utilization of health services and lifestyles. The regional differences may be related to economic development; as we discuss earlier, higher HTN rates are found in urban areas than rural. This is similar to regional gaps, suggesting that the gaps may be caused by economic development. The differences of economic development across regions could influence behavior and lifestyles, medical and health conditions, and other aspects (23). For example, it can lead to poor lifestyles, such as less exercise and eating more foods high in fat, calories, and salt, all of which are personal factors. Besides this, medical conditions and awareness of health care are crucial factors that highly correlate to regional development levels and need to be further improved by the government. Compared with urban areas, rural areas have higher HTN prevalence but lower awareness, treatment, and control in China (18), and the detection rates of HTN in developed areas is significantly higher than that in economically backward areas (24). This further indicates that we should pay more attention to the differences in health care between rural and urban areas. In particular, as shown in our study, the effect of region on urban areas was obviously higher than rural areas, suggesting that regional differences have become more and more critical with the economic development, especially health care. Therefore, in both urban and rural areas, different prevention and control measures should be taken according to their characters and medical conditions. Especially in rural areas, the lower awareness, treatment, and control rates of HTN can be improved by guaranteeing the financial support for medical treatments and improving the utilization level of medical and health services.

## Limitations

Although we have obtained some meaningful results by standardization and decomposition methods, there are several limitations in this study. First, we decomposed and standardized the HTN rates of older adults in urban and rural populations. That is, we divided older adult patients with HTN into urban and rural populations based on the assumption that there are plenty of differences in the population structure between urban and rural areas in China. Yet, if the compositions of populations to compare were similar, the efficiency of this method would be reduced. Second, when more factors are included, the required sample size also increases. In this study, the annual data sample size is not large enough, especially in the beginning years. Therefore, we cannot include more factors in our study.

## Conclusions

By analyzing the HTN rates of older adults in urban and rural China over the past 25 years from 1991 to 2015, we found that the prevalence of HTN in Chinese older adults was on the rise, and the standardization gaps were not as big as crude rates. The urban–rural gaps showed a trend of fluctuation over time; the gaps showed decreasing trends during 1993–1997, then increased during 1997–2011, and declined again. This dynamic change trend was mainly related to the medical security policy formulated by the Chinese government. As for the component contributions, the effects of age, education, and gender on the HTN rates tended to be limited and stable, whereas regional differences had the greatest impact on HTN for both urban and rural older adults in 1991–2015, especially in urban areas. This indicates that the trends of HTN rates and gaps for urban and rural areas mainly come from the variation of regional differences. However, one of the major factors contributing to regional differences in HTN is the medical situation, which needs to be further improved by health policymakers in the formulation of public health policies due to the large gap in medical and health resources between urban and rural areas.

## Data Availability Statement

The data used in the study are openly available in the CHNS website at: https://www.cpc.unc.edu/projects/china.

## Ethics Statement

The studies involving human participants were reviewed and approved by the University of North Carolina at Chapel Hill, and the National Institute of Nutrition and Food Safety and the Chinese Centre for Disease Control and Prevention. The patients/participants provided their written informed consent to participate in this study.

## Author Contributions

JW and QY designed the study. QY drafted the first version. SL and JW revised the manuscript. All authors contributed to the article and approved the submitted version.

## Funding

This work was supported by the major project Healthy Life Expectancy and Health Level Measurement of Population (Grant No.: 17ZDA124) from the National Office for Philosophy and Social Sciences in China. The funders had no role in the study design, data collection and analysis, or preparation the manuscript.

## Conflict of Interest

The authors declare that the research was conducted in the absence of any commercial or financial relationships that could be construed as a potential conflict of interest.

## Publisher's Note

All claims expressed in this article are solely those of the authors and do not necessarily represent those of their affiliated organizations, or those of the publisher, the editors and the reviewers. Any product that may be evaluated in this article, or claim that may be made by its manufacturer, is not guaranteed or endorsed by the publisher.
